# Spike of interstitial PO_2_ produced by a twitch in rhythmically contracted muscle

**DOI:** 10.14814/phy2.14699

**Published:** 2021-01-05

**Authors:** Aleksander S. Golub, William H. Nugent, Bjorn K. Song

**Affiliations:** ^1^ Song Biotechnologies, LLC Baltimore MD USA

**Keywords:** exercise, O_2_, phosphorescence quenching, PO_2_, rhythmical contraction, skeletal muscle

## Abstract

Oxygen (O_2_) exchange between capillaries and muscle cells in exercising muscles is of great interest for physiology and kinesiology. However, methodical limitations prevent O_2_ measurements on the millisecond scale. To bypass the constraints of quasi‐continuous recording, progressive measurements of O_2_ partial pressure (PO_2_) in rhythmically contracting skeletal muscle were compiled to describe the O_2_ kinetics surrounding and including a single muscle contraction. Phosphorescence quenching microscopy measured PO_2_ in the interstitium of the rat spinotrapezius muscle. Measurements were triggered by contraction‐inducing electrical pulses. For the first 60 seconds, measurement preceeded stimulation. After 60, measurement followed with a progressive 20 ms increment. Thus, the first 60 measurements describe the overall PO_2_ response to electrical stimulation initiated after a 10 second rest period, while 61–100 (stroboscopic mode) were compiled into a single 800 ms profile of the PO_2_ transient surrounding muscle contraction. Thirty seconds of stimulated contractions decreased interstitial PO_2_ from a baseline of 71 ± 1.4 mmHg to an “active” steady‐state of 43 ± 1.5 mmHg. The stroboscopic mode compilation revealed an unexpected post‐contractile rise in PO_2_ as a 205 ms spike with a maximum amplitude of 58 ± 3.8 mmHg at 68 ms, which restored 58% of the PO_2_ drop from baseline. Interpretation of this phenomenon is based on classical experiments by G.V. Anrep (1935), who discovered the rapid thrust of blood flow associated with muscle contraction. In addition to the metabolic implications during exercise, the physiological impact of these PO_2_ spikes may grow with an increased rate of rhythmical contractions in muscle or heart.

**New&Noteworthy:**

The principal finding is a spike of interstitial PO_2_, produced by a twitch in a rhythmically contracting muscle. A possible mechanism is flushing capillaries with arterial blood by mechanical forces. A technical novelty is the PO_2_ measurement with a “stroboscopic mode” and progressively increasing delay between stimulator pulse and PO_2_ measuring. That permitted a 20 ms time resolution for a 205 ms spike duration, using an excitation flash rate one per second.

## Graphical abstract

During muscle contraction when local oxygen tension is expected to decrease, we detected a spike (6), which is believed result from a bolus of arteriolar blood flushing the capillaries. This novel finding was made possible by combining phosphorescence quenching microscopy with a “stroboscopic mode” that allows for temporal scanning of oxygen gradients with a millisecond time resolution.

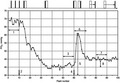

## 1 INTRODUCTION

Muscle cell oxygen (O_2_) consumption increases substantially at the transition from rest to exercise (Behnke et al., 2002; Behnke et al., 2001; Golub et al., 2011), leading to a decrease in the partial pressure of O_2_ (PO_2_) in the interstitial space on the surface of muscle cells and adjacent microvessels (Hirai et al., 2018; Nugent et al., 2016). Blood flow rises to meet this demand on the order of seconds extending to minutes, but the immediate, millisecond responses surrounding contraction have yet to be characterized.

The study of PO_2_ levels associated with variable respiration in muscle cells at rest and exercise became possible with the invention of the phosphorescent quenching method (PQM) for measuring oxygen (Vanderkooi et al., 1987). In recent decades, PO_2_ dynamics in the microvessels responding to rhythmic contractions of the rat spinotrapezius muscle have been characterized (Behnke et al., 2002; Nugent et al., 2016). Because of these works, the rat spinotrapezius muscle has become a standard for studying O_2_ transport in organs with a controlled change in the respiration rate of myocytes. PQM can localize the PO_2_ signal in various physiological compartments of the organ. With intravascular administration of an oxygen probe, PO_2_ can be measured in the plasma of microvessels, locally or in the mass of vessels (Behnke et al., 2001; Golub & Pittman, 2008; Smith et al., 2002; Torres Filho et al., 1996). Situated between the dense capillary network that delivers O_2_ and the skeletal muscle fibers that consume it, the interstitial space is a prime focal point for measuring O_2_ availability.

The rat spinotrapezius muscle is a thin planar organ, which is convenient for bio‐microscopy, thermal stabilization, and isolation from ambient air. The loading of a phosphorescent oxygen probe into the interstitium of a surgically exposed muscle occurs quickly and evenly, which ensures high signal quality for PQM. However, the PQM technique has a temporal limitation in application to a stationary fluid.

For exponential analysis, the phosphorescence signal must have low noise, which is provided by the selective collection of emitted phosphorescence and intense excitation pulse. But, the input of light causes phosphorescent consumption of oxygen, which is proportional to the excitation energy absorbed (Golub & Pittman, 2008). In stationary interstitial fluid, discrete O_2_ depletions may be compensated by diffusion from capillaries and cells, but if the frequency of excitation pulses is too high, it could have a cumulative effect on measurements. The technical parameters of PQM setups are optimized so the method's oxygen consumption is an insignificant fraction of total PO_2_. It was previously estimated that in the muscle interstitium, the rate of excitation pulses should not exceed 5 per second (Golub & Pittman, 2008). While this 200 ms resolution is acceptable for tracking overall PO_2_ dynamics between bouts of exercise and rest, it cannot report the rapid changes in interstitial PO_2_ associated with muscle twitch.

It is known that after the onset of rhythmical contractions, the metabolic situation in the muscle comes to a new “active” steady‐state with a lower microvascular PO_2_ (Behnke et al., 2001; Golub et al., 2011; Nugent et al., 2016). At this new plateau, the stroboscopic principle with a phase rotation can be applied to PO_2_ measurements and, through a series of 1 Hz excitation light flashes, compile a time course of PO_2_ following contraction with a 20‐ms temporal resolution. Three conditions are required: stationary PO_2_ in the contracting muscle, synchronization between contraction and excitation light pulse, incrementing delay of the flash with respect to the onset of the electrical stimulation pulse. We have employed this approach to study the dynamics of interstitial PO_2_ in response to muscle contraction with a high temporal resolution.

## 2 MATERIALS AND METHODS

### 2.1 Animal experiments

SoBran Biosciences Inc. approved the following animal protocol and experimental procedures (IACUC Protocol no. SON‐003‐2018), which were executed by Song Biotechnologies LLC researchers. They are consistent with the National Institute of Health Guidelines for the Humane Treatment of Laboratory Animals, as well as the American Physiological Society's Guiding Principles in the Care and Use of Animals. Six male Sprague Dawley rats (306 ± 10 g; Harlan, Indianapolis, IN, USA) were used in the experiments.

### 2.2 Surgical preparation

Animals were inducted with 1–5% isoflurane in medical air for initial preoperative preparation and cannulations. The femoral vein was then accessed and cannulated with polyethylene tubing (PE‐90) to enable the continuous infusion of anesthetic, alfaxalone acetate (Alfaxan; Schering‐Plough Animal Health, Welwyn Garden City, UK), at rate ~0.1 mg/kg/min). Continuous infusion, with responsive adjustment to animal reflexes, heart rate, and oxygen saturation indicators, provided a steady plane of anesthesia through the conclusion of surgical preparation and measurement. A tracheal tube was inserted to maintain a patent airway. A femoral artery cannula was connected to a pressure transducer for monitoring systemic circulatory variables with a multichannel physiological monitoring system (BIOPAC MP‐150; BIOPAC Systems, Goleta, CA, USA). The animal core and exteriorized spinotrapezius muscle temperatures were maintained by a custom animal platform (Golub & Pittman, 2003), and a rectal probe monitored the core temperature. The main parameters characterizing a physiological status in six rats were: mean arterial pressure 117 ± 4 mmHg; heart rate 433 ± 8 min^−1^; body temperature 37 ± 0.2°C. Following the completion of experimental measurements, animals were euthanized with Euthasol (360 mg/ml of pentobarbital and 50 mg/ml of phenytoin sodium administered I.V. at 3 ml/kg; VetOne; Boise, ID).

### 2.3 Spinotrapezius muscle preparation

The rat spinotrapezius muscle surgical preparation was similar to the original descriptions (Gray, 1973) with some modification to measurements of interstitial PO_2_ in isometrically contracting muscle. The exteriorized muscle was placed on a trans‐illuminated pedestal of the 3‐D printed animal platform, thermo‐stabilized at 37°C. The edges of the muscle were fixed with seven sutures to a rigid frame to minimize muscle movement for isometric contractions. Two chlorinated silver wire electrodes were attached along the side edges of the muscle for electrical stimulation. A bout of electrical stimulation (1–5 s) was applied at the end of the preparation procedure to ensure proper muscle fixation and electrode connection.

The muscle was allowed to stabilize for 20 minutes, while the oxygen probe loaded into the interstitium (Golub et al., 2011). The phosphorescent oxygen probe employed in our measurements was a Pd(II) meso‐Tetra(4‐carboxyphenyl)porphine (PdT790; Frontier Scientific, Newark, DE) conjugated to bovine serum albumin as previously described (Golub & Pittman, 2016; Vanderkooi et al., 1987). The probe solution, at concentration 8–10 mg/ml of Pd‐TCPP, was directly applied to surgically opened muscle for 20–30 min and then blotted with a filter paper. The muscle was covered with the gas barrier film Krehalon, CB‐100 (Kureha Corporation, Tokyo, Japan).

An objective‐mounted, transparent (Krehalon) airbag was inflated to provide organ compression at a pressure of 8 mmHg. Low‐pressure surface contact allowed free blood circulation, while providing a tight seal of the gas barrier film to the muscle surface. The pressure was necessary to prevent the accumulation of a fluid layer between the film and tissue and to exclude a convective transfer of oxygen into the region of measurement due to muscle contraction.

### 2.4 Intravital microscopy and PQM

Measurements of interstitial PO_2_ were carried out using an Axioimager‐A2m microscope with a 20X/0.8 Plan‐Apochromat objective lens (Carl Zeiss, Germany). The measurement technique described in detail in our previous publications (Nugent et al., 2016) had several modifications to upgrade its performance. The excitation light source was a 520 nm green laser diode (NDG7475 1 W; Nichia Tokushima, Japan) powered by laser driver iC‐HKB (www.ichaus.com). An optical cube in the path of the epi‐illumination train contained a dichroic beam splitter (567 nm DMLP Longpass; Thorlabs, Newton, NJ, USA) and a wide‐band, interference filter (Longpass Cut‐on >650 nm; Thorlabs) for emitted phosphorescence light.

The circular (diameter 450 µm) epi‐illuminated area covered more than ten fiber's width (33.6 ± 0.7 µm; 22 measurements). The beam area was limited by a field diaphragm to employ the most homogeneous central part of the beam. The laser pulse duration was set to 1 µs, and the energy of a single pulse delivered to the muscle surface was 8 pJ/µm^2^. With the combination of a low probe concentration and intensity of excitation illumination, oxygen consumption by phosphorescence quenching reduced PO_2_ measurements by a minimal 0.4% per excitation flash, as previously described (Golub et al., 2018).

The phosphorescence decay curve was detected with a photomultiplier tube R9110 and a socket C12597‐01 (Hamamatsu Photonics, Japan). The detector was assembled in a PXT‐1 housing (Thorlabs) mounted on the microscope upper port. The current output of the detector was converted into a voltage signal with the precision operational amplifier LT1028. The initial segment of the phosphorescence decay signal containing the excitation flash and fast fluorescence transients was truncated by disabling the amplifier output for 5 µs with an analog switch ADG419. The phosphorescence decay analog signal was sampled at 200 kS/s and digitized with 16‐bit accuracy using the software LabView and hardware NI PCIe‐6361 board (National Instruments, Austin, TX, USA). The duration of the recorded phosphorescence decay curves was 2 ms with 400 data points collected per curve. The period starting from excitation flash and ending when a decay curve data is collected is a PO_2_ measurement time interval.

The principle of exponential analysis of the phosphorescence decay curves and fitting model for recovery of a mean PO_2_ from heterogeneous decay curves is as previously described (Golub & Pittman, 2016; Golub et al., 1997). The computer program for data acquisition, processing of curves, and the calculation of PO_2_ during the measurement procedure were composed in‐house as a Virtual Instrument in LabView.

### 2.5 Muscle stimulation and location of sites

The built‐in electrical stimulator produced a symmetrical 10 volt and 20 ms duration pulse delivered at 1 Hz to a pair of chlorinated silver wire electrodes placed alongside the muscle. The stimulator circuit was optically isolated from other electronic units.

Measurement sites were clustered in the central part of the muscle, where contraction displacement was minimal. Excitation pulses were targeted to a 450 µm circular muscle region between transversal arterioles so that the vessels were represented mainly by capillaries and interstitial PO_2_ values belonged to the interface between the capillaries and muscle cells.

### 2.6 Experimental procedure

**FIGURE 1:**
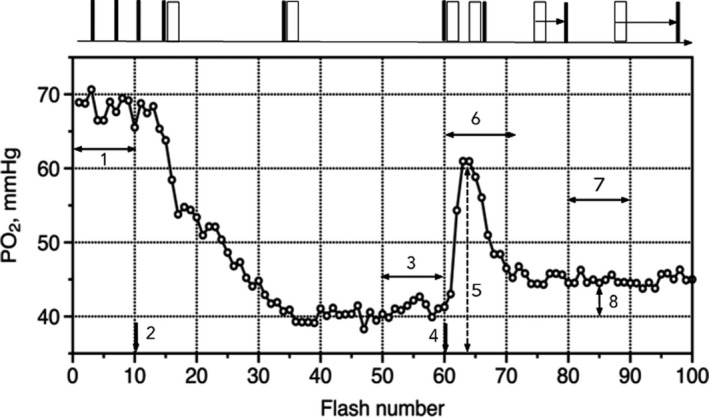
Protocol diagram and example of a recorded PO_2_ trace obtained in accordance with the experimental protocol. PO_2_ is plotted versus the number of the excitation flash, which corresponds to the time in seconds. However, data after measurement #60 no longer reflect continuity in time due to the incrementing delay of PO_2_ measurements. The top diagram shows the arrangement between the PO_2_ measurement time period (2 ms, black bars) and the pulse of electrical stimulation (20 ms, white bars). The first 10 seconds were taken to measure PO_2_ without stimulation, then starting from #11 and up to #60 inclusive, the PO_2_ measurement period immediately followed by the stimulator pulse. Starting from #61 the PO_2_ measurement period followed the stimulation pulse with an increment of 20 ms for every next pulse (stroboscopic mode). That permitted the reconstruction of the PO_2_ transient with time resolution 20 ms at a real flash rate of less than 1 per second. The bottom graph shows the result of PO_2_ measurements during the experimental procedure: (1) baseline PO_2_ as a mean of #1‐10 values; (2) start of 1 Hz electric stimulations from #11 to #100; (3) averaged PO_2_ values #51‐60 as a characteristic of an active steady‐state PO_2_ in rhythmically contracting muscle; (4) start of the stroboscopic mode of PO_2_ measurements #61‐100; (5) amplitude of the PO_2_ peak induced by muscle contraction; (6) duration of the PO_2_ peak considering 20 ms intervals between data points; (7) averaged PO_2_ values #81‐90 to characterize the (8) trend to post‐contractile steady‐state PO_2_.

Event timing during the experiment was governed by a micro‐controller board Arduino Uno R3 (https://www.arduino.cc/), according to a sketch illustrated by the top diagram in Figure 1. Interstitial PO_2_ was measured in a selected site of the muscle for 100 seconds and divided into one second time intervals, from #1 to #100. Each PO_2_ measurement interval (2 ms duration, shown as a black bar on the top diagram Figure 1) started with an excitation flash, so the total set of obtained 100 PO_2_ values was plotted versus the flash number, not versus time in seconds to avoid complications after measurement #60 when the intervals between measurements increment for 20 ms every second. With this in mind, the number of a flash is the same as for the corresponding one second time interval.

Measurements began after animal recovery and stabilization from surgery. Each measurement series (100 PO_2_ samples) was preceded by at least 4 minutes of rest after the previous bout of stimulated twitches. The first 10 PO_2_ values (#1‐10 s) were recorded without electrical stimulation and averaged as a baseline PO_2_ (Figure 1). Starting from flash #11 to #60, the electrical stimulation pulse (20 ms duration, presented as a white bar on the top diagram Figure 1) occurred after the PO_2_ measuring flash (black bar), so the PO_2_ was sampled about a second after stimulation and twitch of the muscle. Stimulated muscle contractions resulted in a decrease in interstitial PO_2_, which stabilized after approximately 30 seconds/contractions. Ten values of PO_2_ for seconds #51‐60 were averaged as an active steady‐state PO_2_.

**FIGURE 2:**
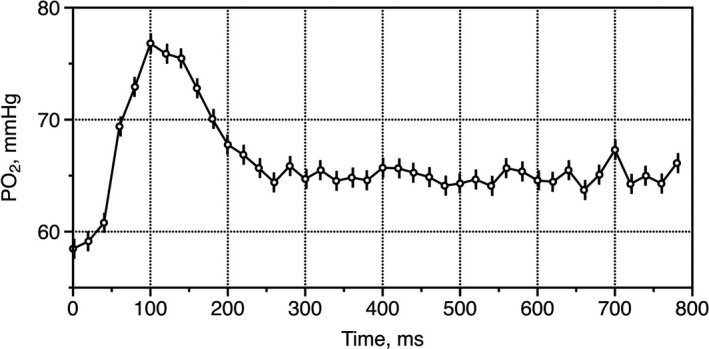
Compilation of the PO_2_ profile following the 20 ms stimulation pulse. The dots represent the mean PO_2_ values for five records made in the same muscle site, and the vertical lines represent SE. The graph of 40 PO_2_ values from #60 through 100 is plotted against time (ms), corresponding to the fast interstitial PO_2_ spike following muscle stimulation. The duration of this spike is about 260 ms, and the total reconstruction is 800 ms.

The pattern of flash preceding a stimulating pulse recorded data that is presented in real‐time and lasted until measurement #60 (see the top diagram in Figure 1). Measurement #60 was taken as the zero value for the PO_2_ transient that followed the stimulation pulse. The next measurement of PO_2_ (#61) followed the stimulating pulse, that is, had a delay of 20 ms relative to the front of the stimulating pulse. This delay increased an additional 20 ms with every subsequent flash reaching 800 ms by the end of the recording. Thus, respective to the front of the stimulation pulse, point #61 had a 20 ms delay, #62 had a 40 ms delay, and #63 a 60 ms … up to 800 ms at flash #100. Since an active steady‐state of PO_2_ was established during measurements #50‐60, it was assumed that muscle status was maintained until measurement #100. This “stroboscopic mode” with the incremental time shift was compiled to produce a 0.8 s profile of the changes in PO_2_ associated with single muscle contraction with a time resolution of 20 ms, without exceeding the actual flash rate of 1 per second (Figure 2).

The amplitude and duration of the PO_2_ transient were compiled by the set of incremental measurements #61‐100 with the time intervals between adjacent values of 20 ms. Some of the compiled PO_2_ tracks had a slow positive trend in PO_2_ after reaching a stationary state, which exceeded the active stationary level (#51–60). We used the average value of the “post‐contractile” steady‐state PO_2_ (#81–90) to characterize this effect.

### 2.7 Statistics

In each of the six muscles, 4–5 PO_2_ records were carried out in 3–4 tissue sites; a total of 21 sites were explored. The number of PO_2_ records made in each muscle was 15–19; a total of 101 records were obtained. A Descriptive Statistics and Paired t‐test were used from the Data Analysis Tools in Microsoft Excel (Microsoft Corporation, Redmond, WA, USA). All data are presented as mean ± SEM (number of measurements). Diagrams were built with a graphical tool DataGraph 4.4 (http://www.visualdatatools.com/DataGraph/).

## 3 RESULTS

**FIGURE 3:**
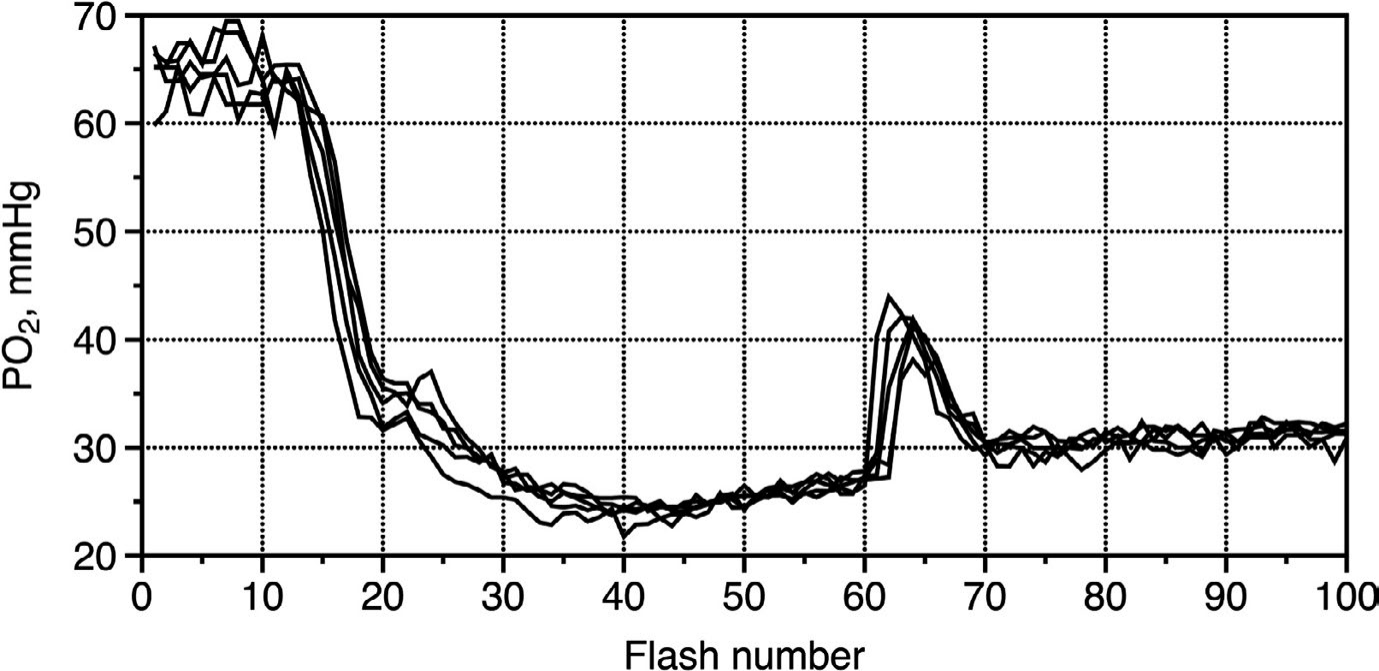
Five PO_2_ records made in the same site in the muscle. A four‐minute rest interval between records was sufficient to restore the baseline level of PO_2_ (#1–10). The stroboscopic mode started at flash #60, so the following lines demonstrate the PO_2_ changes with step 20 ms. Eleven values of PO_2_ (#60–70) covered the entire PO_2_ spike, lasting approximately 220 ms. After 30 seconds of stimulation, pre‐contraction PO_2_ reached a minimum, and then a small positive trend appeared, likely due to the slow development of exercise hyperemia.

The PO_2_ time courses showed a high degree of reproducibility between different tissue sites and animals (Figure 3). A 4–5 min rest between experimental recordings was sufficient to restore the baseline PO_2_, which, averaged over all experimental records, was 71 ± 1.4 mmHg (flash #1–10, Table 1). The onset of 1 Hz electrical stimulation after flash #11 caused a gradual decrease in interstitial PO_2_ until it reached an active steady‐state of 43 ± 1.5 mmHg, in about 30 s. The average difference in PO_2_ between the baseline and active steady‐state PO_2_'s (PO_2_ span) was 29 ± 1.2 mmHg (*p* < 0.001, Table 1).

**TABLE 1:** Statistical data for interstitial PO_2_ measured in the spinotrapezius muscle. The total number of studied muscles was 6. PO_2_ was recorded in 21 sites and the total number of experimental recordings of PO_2_ was 101. The meaning of the measured parameters is illustrated and described in Figure 1.

N = 101	Mean ± SEM	Units
Baseline PO_2_ (#1‐10)	71.3 ± 1.4	mmHg
Active steady‐state PO_2_ (#50‐59)	42.5 ± 1.5	mmHg
PO_2_ span	28.8 ± 1.2	mmHg
Post‐contractile steady‐state (#81‐90)	46.2 ± 1.5	mmHg
PO_2_ change between steady‐states	4.1 ± 0.4	mmHg
Peak PO_2_	57.6 ± 1.8	mmHg
Peak PO_2_, % of span	58.0 ± 3.8	%
Spike duration	204.8 ± 6.3	ms
Peak delay	68.7 ± 3.3	ms

Incremental PO_2_ data from the stroboscopic mode were used to reconstruct the 800 ms following the front of the electric stimulator pulse. This profile revealed that muscle contraction produced a substantial and rapid increase in interstitial PO_2_. The maximum value was reached at an average of 68.7 ± 3.3 ms, followed by a decline to a post‐contractile steady‐state. The total duration of the PO_2_ spike from the beginning of the stimulating pulse to the decrease of PO_2_ to the active steady‐state level was 204.8 ± 6.3 ms. The amplitude of the PO_2_ peak was 57.6 ± 1.8 mmHg, 15.1 mmHg above the active steady‐state PO_2_ level (#51‐60, see Figure 1). That difference was significant (*p* < 0.001) and amounted to 58% of the PO_2_ span between baseline and active steady‐state (Table 1, Figure 1).

An essential condition for this technique is to achieve stationarity, that is, the identity of all tracks of PO_2_ after each stimulating impulse. This condition was well satisfied for the spike, but a slow increase in PO_2_ was observed at the tail end. In many recordings, an active steady‐state had a small positive trend (Figure 3), but on average, the difference between two stationary PO_2_ values, active and post‐contractile, was 4.1 ± 0.4 mmHg and was not statistically significant.

## 4 DISCUSSION

The central finding of this experimental study is the spike of interstitial PO_2_ that follows muscle contraction. An explanation of this phenomenon's mechanism is possibly found in the results of classical studies by G.V. Anrep (Anrep & Saalfeld, 1935). In this work, the inflow and outflow of blood from a contracting muscle were recorded using a fast‐acting anemometer. During stimulated muscle contraction, interruption and “back thrust” of inflow occurred, while the venous outflow increased several times over a spike of 200 ms. Spike magnitude did not depend on muscle load but the intensity of the electric stimulus. This spike of venous blood flow during contraction was confirmed by more modern methods (Dobson & Gladden, 2003). Our PO_2_ measurements were made in the interstitial fluid exclusively surrounding capillary networks. If we assume that at the time of muscle contraction, a bolus of blood is squeezed from arterioles through the capillaries and into venules, then the resultant fast enrichment of the pericapillary fluid with oxygen is responsible for the observed increase in PO_2_.

Blood flow is the primary indicator of the interstitial O_2_ supply and is often regulated by the arteriolar diameter. In exercising muscle, arterioles dilate to increase blood flow through the capillary beds, which are the major sites of gas exchange. The 50 second period of 1 Hz contractions preceeding the switch to stroboscopic mode was designed to prevent changes in vascular resistance from confounding the 800 ms contraction profile, which is confirmed by reaching satisfactory equilibrium of the active steady‐state. To our knowledge, there are no existing tracings of PO_2_ in response to contraction on the millisecond time scale. Thus, our finding is novel. Where our studies do overlap those of other laboratories is with the development of the active steady‐state in response to rhythmic contractions, and our #1–60 profile is in agreement (Hirai et al., 2018; Poole et al., 2008; Poole & Ferreira, 2007).

One cannot conflate the circulatory phenomenon described by G.V. Anrep in a contracting muscle with the lately debated “muscle pump” theory (Folkow et al., 1970; Hamann et al., 2003). The mechanics of that hypothetical muscle pump are based on compression of the veins equipped with the anatomical venous valves. There are no valves in the microvasculature of the spinotrapezius muscle, and another explanation is needed for unidirectional blood flow during muscle compression. Possibly, the big difference between arterioles and venules in vascular resistance and blood pressure ensures the rectification of blood flow caused by a rapid increase of intramuscular pressure.

Following the muscle contraction‐induced decrease in interstitial PO_2_ (29 mmHg), the increase in PO_2_ closes the gap between active steady‐state and baseline by 58% for 200 ms, which amounts to an absolute increase in oxygenation of less than 10%. The contribution is modest but noteworthy since it occurs at a moment of high oxygen demand for ATP regeneration. However, this contribution may be more significant in higher frequency and intensity rhythmic contractions. At five and more contractions per second, these 200 ms PO_2_ spikes may cumulatively provide a more substantial fraction of oxygen supply, working in concert with local oxygen capacitance in myoglobin, acting as a smoothing filter for pulsing PO_2_. Rhythmically contracting muscles, like myocardium, especially in small animals with high heart rates, may use this mechanism as a vital source of O_2_. Additionally, this effect may contribute to the oxygen supply in the tachycardic human heart.

The small positive trend in PO_2_ that appeared after 30 seconds of serialized contractions was probably due to the slow development of exercise hyperemia. This trend continued during the stroboscopic mode measurements, but it had a low magnitude, which was not significant and did not affect the detection of the PO_2_ spike.

## 5 CONCLUSIONS

After the onset of stimulated, rhythmical contraction of the rat spinotrapezius muscle, the interstitial PO_2_, observed at a 1 Hz resolution, generally decreases to an active steady‐state level. However, at higher temporal resolutions, it is observed that each muscle contraction is accompanied by a spike of interstitial PO_2_, which indicates the forced flush of arterial blood through a capillary network. The contribution of the additional oxygen delivered through that mechanism may become higher with increased frequency of contraction and intensity of stimulation. The impact of this phenomenon on oxygen supply in a heart muscle at high heart rates is of great interest.

## DISCLOSURE

No conflicts of interest, financial or otherwise, are declared by the authors.

## AUTHOR CONTRIBUTIONS

A.S.G. and B.K.S. conceived and designed the research; A.S.G. and B.K.S. performed the experiment. A.S.G., W.H.N., and B.K.S. analyzed the data; A.S.G., W.H.N., and B.K.S. interpreted the results of experiments; A.S.G. prepared the figures; A.S.G., W.H.N. drafted the manuscript; A.S.G., W.H.N., and B.K.S. edited and revised the manuscript; A.S.G., W.H.N., and B.K.S. approved the final version of the manuscript.
